# How to Reduce Food Waste Caused by Normative Illusion? A Study Based on Evolutionary Game Model Analysis

**DOI:** 10.3390/foods11142162

**Published:** 2022-07-21

**Authors:** Mengling Tian, Yangyang Zheng

**Affiliations:** Business School, Wenzhou University, Wenzhou 325035, China; mltian0817@163.com

**Keywords:** food waste, normative illusion, evolutionary game, stakeholders

## Abstract

Reducing food waste is a priority for all sectors of society as it threatens national food security and the sustainability of global agriculture. Many studies on food waste have focused on a single subject, and the psychological factors of consumer waste are often overlooked. Based on evolutionary game theory, this paper introduces consumers’ normative illusion, constructs an evolutionary game model in which the government, caterers and consumers collaborate to reduce food waste, and simulates and analyses the behavioural strategies of the three stakeholders. The results show that: Firstly, food waste can be reduced under certain conditions by incentive-guided and punishment-inhibited policies. Moreover, incentive-guided policies can reduce government expenditures more than punishment-inhibited ones. Secondly, implementation of prior intervention, the resultant intervention and reducing the probability of consumers’ aversion to the intervention of caterers can optimise the government’s punishment-inhibited policy. Finally, under the punishment-inhibited policy, caterers can bear 60% of the prior intervention costs for food waste management. When caterers invest 40–60% of the prior intervention costs, both caterers and consumers can achieve the ideal state of cooperation; caterers can accept 40% of the resultant intervention cost for food waste management, and when the resultant intervention cost is less than 40%, consumers choose not to waste. Both caterers and consumers are involved in reducing food waste when the probability of consumer dissatisfaction with a caterer’s intervention is reduced to less than 40%.

## 1. Introduction

Food is related to the national movement and people’s livelihood, and food security is an important foundation for national security. In 2021, China’s grain production reached 1365.7 billion catties, a bumper harvest for 18 consecutive years, but the phenomenon of food waste is equally astounding [1]. It is estimated that China’s urban catering food waste amounts to about 17–18 million tons, with each person wasting about 93 g of food per meal, which is equivalent to dumping the food rations of 200 million people [2]. Food waste is a serious threat to the resource environment [3,4,5], economic development [6,7] and food security [8,9]. At present, under the background of various crises, such as the global novel coronavirus pneumonia and natural disasters, reducing food waste is an urgent problem that needs to be solved worldwide.

The Chinese government attaches great importance to food waste. Chinese President Xi Jinping has repeatedly stressed the need to eliminate restaurant waste, and advocated the establishment of a social trend of “strict economy, anti-waste”. In 2013, the Chinese government issued the “Regulations on Implementing Economy and Opposing Waste in Party and Government Organizations” which spearheaded a cost-cutting campaign against extravagance and waste within the party and government organisations. In 2014, the “Opinions on Practicing Conservation and Combating Food Waste” was issued, which pointed out that it is necessary to eliminate the waste of official meals, encourage frugal meals in unit canteens, and promote a scientific and civilised catering consumption model. In 2021, the Anti-Food Waste Law of the People’s Republic of China was officially implemented, which legally restricts the food waste behaviour of government organisations, catering service operators, schools, catering takeaway platforms and other actors. China’s food waste policy targets have shifted from government organisations to the general public. However, as the economy grows and people’s living standards improve, the proportion of consumers eating out continues to rise and catering waste still occurs in China [10].

Food waste has been studied in the literature in terms of food waste measurement, food waste subjects and actions to reduce food waste. Food waste is mostly quantified by weighing or estimating, and the most common method is to estimate the percentage of food waste [11,12,13]. However, the results of the measure need to be further explored as there is insufficient statistical evidence on the percentage of waste data. Some scholars have also measured food waste in terms of energy equivalents [6], value [14] and resource and environmental costs [3]. As the subject of food waste, factors, such as consumers’ education level, perceived attitudes, ability to manage food, household structure and income level all impact on their food waste behaviour [15,16,17,18]. Some scholars have also attempted to explore food waste from the perspectives of social norms and cultural factors [19,20]. Reducing food waste can be implemented at three levels: government, society and individuals. Clear government legislation and policies can effectively reduce food waste [21]. For example, the United States guides social subjects to donate food to non-profit organisations by reducing the cost of food donations and offering tax relief for companies that donate [22], while France focuses on punishment policies, such as increasing penalties for food waste and forcing restaurants to provide customers with environmentally friendly packaging bags [23]. Civil society organisations are important agencies in managing food waste, with research showing that Australian organisations can reduce food waste by $5.71 for every $1 invested [24]. Raising consumer awareness of the amount of food waste and providing relevant technical training can enable consumers to reduce food waste [25].

These studies provide a solid theoretical basis for this paper, but it also has the following shortcomings. First, there is less research on the psychology of food waste, and few studies have found that consumers’ normative illusion is a contributing factor. Second, most existing studies have analysed food waste behaviour on only one subject, and few have incorporated multiple subjects into the same system. Third, the studies have not looked at how to reduce consumer food waste behaviour through the intervention of caterers. China has a distinctive “shame culture” on the dining table compared to other countries. It is manifested in the extravagance and waste of the banquet host, entertaining guests with a catering scale that far exceeds the number of guests to show their identity and status [26]. “Shame culture” has become a valuable yardstick for judging interpersonal relationships among groups and has spawned unhealthy and immature social consumption psychology, such as “conformity consumption”, “conspicuous consumption” and “comparison consumption” [27]. This psychology creates a normative illusion among individual consumers, who misjudge wasteful behaviours such as ‘loving ostentation and saving face’ as being in line with social norms. However, very little research has been conducted in this area.

To fill these research gaps, this paper constructs a tripartite evolutionary model of the government, caterers and consumers based on a Chinese scenario. We focus on answering the following three questions. First, what is the evolutionary stable state (ESS) in the dynamic replication system composed of the government–caterers–consumer, and what are the key factors affecting the ESS? Second, what are the comparative effects and optimal implementation of different government policies? Third, what is the effect of the intervention behaviour of caterers on reducing consumer waste? What is the optimal level of intervention? The main contributions of this paper are as follows: First, we propose a new collaborative development framework that integrates the government, caterers and consumers into a complex system. By observing the effect of their interaction mechanism on reducing food waste, we analyse the conditions for reaching an ideal steady state. Second, we simulate the two main policies implemented by the current government, compare the effects of the two policies on reducing food waste and provide a theoretical basis for the government’s policy formulation. Third, the effectiveness of the three interventions by caterers is compared in terms of the wasteful behaviour of consumers influenced by the illusion of norms.

The remainder of this article is structured as follows: Section 2 describes the research problem and constructs a tripartite evolutionary model about the government, caterers and consumers. Section 3 shows the evolutionary game’s modelling process and analyses the three parties’ evolutionary equilibrium and system stability point. Section 4 simulates different government policies and optimises the government’s punishment and containment policies through key influencing factors. Section 5 summarises the main conclusions of this paper, puts forward policy recommendations accordingly and analyses the limitations of the paper and the outlook for future research.

## 2. Model Building

### 2.1. Theoretical Basis

This paper studies the problem of reducing food waste using an evolutionary game theory approach. The evolutionary game theory combines evolutionary thinking with game theory and uses the systems of biological disciplines to study the entire economy and society. Unlike classical game theory, evolutionary game theory not only takes into account the bounded rationality of players, but also provides a powerful framework for examining changes in beliefs and norms over time and predicting outcomes of competing strategies in dynamic environments [28]. In recent years, evolutionary game theory has been increasingly used in studying population competition strategies, especially in energy and environmental governance [29]. For example, Luo and Zhao [30] developed a game model between the government and supermarkets to determine how to implement PAFW more effectively. They suggested that the government should make policy improvements to support supermarkets in implementing PAFW and reduce the cost of the implementation. Zhu, Fan [31] constructed a tripartite evolutionary game model comparing fixed and spot-check penalties for government departments, catering companies and waste disposal companies. It was found that the government could manage food waste in urban areas better with a spot-check penalty scheme while keeping regulatory costs reasonable and promoting resource utilisation. This study is especially relevant to evolutionary game theory because it can be used to analyse the factors that influence the formation of social habits, norms and institutions.

### 2.2. Problem Description

“Banquet etiquette, wine table culture” and other social cultures have spawned uncivilised consumption behaviours, such as comparative consumption, extravagant consumption and conspicuous consumption, and caterers have led customers to over-order to increase their turnover, resulting in serious food waste in China. China wastes 17–18 billion kilograms per year in urban dining, with a per capita rate of 11.7% and 38% of food at large events. [32]. Dining table waste has gradually become a hidden threat that threatens food security, consumers resources and the environment, and destroys a social atmosphere. The Chinese government has taken various steps to curb consumer food waste, including legislation, initiatives and government-enterprise cooperation. Generally speaking, it mainly includes two policies: incentive-guided and punishment-inhibited. In 2021, the Chinese government promulgated and implemented the Anti-Food Waste Law, requiring caterers to strictly implement the anti-food waste system and norms, not to induce consumers to over-order food, and to advocate for caterers to intervene in excessive consumption of customers through publicity, rewards and punishments [33]. According to the fieldwork, the three main ways Chinese caterers intervene in customer over-consumption include prior intervention, resultant intervention and food environment intervention, which indicate messaging through publicity and prompts, rewarding and punishing consumers and reducing plate size, respectively. However, the decision to intervene is largely based on cost-benefit considerations. Although reducing table waste can save food waste disposal costs for caterers, increased customer consumption can increase the business revenue of caterers. In reality, many caterers are reluctant to enforce anti-waste policies because their cooperation with anti-waste policies can turn customers to disgust.

Social diet influences consumer consumption and reducing food waste is typical collective behaviour. Because social norms exist at both the collective and individual level [34], consumers may still have a normative illusion of overestimating the food waste of others and taking pride in it, despite the government’s promotion of a healthy and civilised diet. Under this normative illusion, the non-wasteful behaviour of individual meals is not recognised, while the wasteful behaviour is favoured. In short, the government is faced with a policy dilemma, and catering companies may rashly arouse customer disgust. Consumers’ decision making under the influence of food customs and consumption culture affects the decision making of the government and caterers and, in turn, is influenced by the government and caterers.

### 2.3. Model Assumption

Based on the current situation and policies of food waste in China, the assumptions about the actions and benefits of the participants in the evolutionary model are as follows:

Assumption 1. The government, caterers and consumers are all bounded rationality, and have continuous learning abilities. In the game process, they constantly adjust their strategies according to limited information and expected benefits until they stabilise at the optimal strategy.

Assumption 2. The government can choose an incentive-guided or punishment-inhibited policy, and the implementation probabilities of the two policies are *x* and 1 − *x*, respectively. Caterers can choose to implement the intervention or the non-intervention. The probabilities are *y* and 1 − *y*, respectively. Consumers can choose not to waste or to waste, with probabilities *z* and 1 − *z*, respectively.

Assumption 3. The social benefit brought by the government’s incentive-guided policy, $E_{g1}$; αA, is rewarded for the intervention behaviour of caterers, and α is the government’s incentive intensity. When the government chooses punishment-inhibited policy, it needs to pay the supervision cost βH, and the social benefit obtained from it is $E_{g2}$. The government’s punishment for the non-intervention behaviour of caterers is βF, and β is the government’s supervision intensity. 

Assumption 4. Caterers needs to pay for information transmission and prompting fees μc to implement prior intervention, where μ is the intensity of prior intervention. The implementation of resultant intervention by caterers will punish consumers for wasteful behaviour εf and reward consumers for non-wasteful behaviour εi, where ε is the intensity of the resultant intervention of caterers. The implementation of food environmental intervention by caterers can save the cost of kitchen waste disposal in caterers e. The intervention of caterers may cause disgust among customers, in which the probability is p and the loss caused to the caterers is ps. However, intervention behaviours can bring environmental reputation benefits to caterers E.

Assumption 5. Without intervention, consumers who choose waste have a normative illusion that diminishes with the intensity of prior intervention. Supposing the consumer’s norm illusion benefit is W, if the caterers intervene, the consumer’s norm illusion benefit is (1−μ)W. The government establishes a correct food consumption concept for the public through incentive-guided policy and punishment-inhibited policy. If consumers choose not to waste, the positive benefits brought to the government by the correct consumption concept established by different policies are $I_{1}$ and $I_{2}$, respectively. If consumers choose to waste, the correct consumption concept will bring a negative benefit to them. Consumer waste leads to increased costs for the government to maintain food security and manage the environment G.

Based on the above assumptions, the mixed strategy game matrix of the government, catering companies and consumers is shown in Table 1. The specific variable symbols and meanings are shown in the appendix.

## 3. Model Analysis

### 3.1. Expected Payoff and Replicator Dynamics Equation of Each Participant

According to the payoff matrix in Table 1, it is assumed that $U_{ij}$ and $\bar{U_{i}}$ represent the participants’ expected payoff and average payoff, respectively. i=1,2,3 represent the government, caterers and consumers, respectively, and j=1,2 represent two different decisions of the participants. The expected benefits of different choices for the government, caterers and consumers are as follows:

(1) $$U_{11}=-y\alpha A-(1-z)G+E_{g1}$$

(2) $$U_{12}=-\beta H+(1-y)\beta F-(1-z)G+E_{g2}$$

(3) $$U_{21}=-\mu c-ps+e+E-z\varepsilon i+(1-z)\varepsilon f+x\alpha A$$

(4) $$U_{22}=-(1-x)\beta F$$

(5) $$U_{31}=y\varepsilon i-y(1-\mu )W-(1-y)W+xI_{1}+(1-x)I_{2}$$

(6) $$U_{32}=-y\varepsilon f+y(1-\mu )W+(1-y)W-xI_{1}-(1-x)I_{2}$$

According to the above formula, the average expected return of the three participants can be obtained as follows:

(7) $$\bar{U_{1}}=xU_{11}+(1-x)U_{12}$$

(8) $$\bar{U_{2}}=yU_{21}+(1-y)U_{22}$$

(9) $$\bar{U_{3}}=zU_{31}+(1-z)U_{32}$$

According to the expected returns of the three participants, the replication dynamic equation is calculated as follows:

(10) $$F(x)=\frac{dx}{dt}=x(U_{11}-\bar{U_{1}})=x(1-x)[(E_{g1}-E_{g2})+\beta H-y\alpha A-(1-y)\beta F]$$

(11) $$\begin{array}{r} F(y)=\frac{dy}{dt}=y(U_{21}-\bar{U_{2}})=y(1-y)[-\mu c-ps+e+E-z\varepsilon i+\\ \left(1-z)\varepsilon f+x\alpha A+(1-x)\beta F\right] \end{array}$$

(12) $$\begin{array}{r} F(z)=\frac{dz}{dt}=z(U_{31}-\bar{U_{3}})=z(1-z)[y\varepsilon i+y\varepsilon f-2y(1-\mu )W\\ -2(1-y)W+2xI_{1}+2(1-x)I_{2}] \end{array}$$

### 3.2. Stability Analysis of Evolutionary Game

#### 3.2.1. Stable Strategy Analysis for Each Participant

When the replication dynamic equation is equal to 0, it means that (x, y, z) no longer changes with time, and the choice of each participant is the optimal strategy. According to the differential equation stability principle, when the replica dynamic equation is 0, and its first derivative is less than 0, the replica dynamic system reaches a stable state. Therefore, the stable analysis of the strategies of the government, catering companies and consumers is as follows:

For the government, the following conclusions can be drawn from Equation (10):

(1) When $\frac{E_{g1}-E_{g2}+\beta H-\beta F}{\alpha A-\beta F}<y<1$, then ${\frac{d\left(F\left(x\right)\right)}{dx}|}_{x=1}>0$, ${\frac{d\left(F\left(x\right)\right)}{dx}|}_{x=0}<0$, it can be inferred that x=0 is the evolutionary stable point of the government. It shows that the government has changed from implementing incentive-guided policy to implementing punishment-inhibited policy, and finally stabilised by choosing to implement punishment-inhibited policy.
(2) When $y=\frac{E_{g1}-E_{g2}+\beta H-\beta F}{\alpha A-\beta F}$, then F(x)≡0. It shows that the government chooses the punishment-inhibited policy and the incentive-guided policy to have the same benefits. All x are evolutionary stable, and the policy choice does not change with time.
(3) When $0<y<\frac{E_{g1}-E_{g2}+\beta H-\beta F}{\alpha A-\beta F}$, then ${\frac{d\left(F\left(x\right)\right)}{dx}|}_{x=1}<0$, ${\frac{d\left(F\left(x\right)\right)}{dx}|}_{x=0}>0$, it can be inferred that x=1 is the evolutionary stable point of the government. It shows that the government has changed from implementing punishment-inhibited policy to implementing punishment-inhibited policy, and finally stabilised in choosing to implement the policy of incentive and guidance.

For caterers, the following conclusions can be drawn from Formula (11):

(1) When $\frac{-\mu c-ps+e+E-z\varepsilon i+\left(1-z\right)\varepsilon f+\beta F}{\beta F-\alpha A}<x<1$, then ${\frac{d\left(F\left(y\right)\right)}{dy}|}_{y=1}>0$, ${\frac{d\left(F\left(y\right)\right)}{dy}|}_{y=0}<0$, it can be inferred that y=0 is the evolutionary stable point of caterers. It shows that the caterers changed from intervention to non-intervention, and finally stabilised in choosing the strategy of non-intervention.
(2) When $x=\frac{-\mu c-ps+e+E-z\varepsilon i+\left(1-z\right)\varepsilon f+\beta F}{\beta F-\alpha A}$, then F(y)≡0. It shows that the caterers choose to intervene and do not have the same benefit. All y are in an evolutionary stable state, and the policy choice does not change with time.
(3) When $0<x<\frac{-\mu c-ps+e+E-z\varepsilon i+\left(1-z\right)\varepsilon f+\beta F}{\beta F-\alpha A}$, then ${\frac{d\left(F\left(y\right)\right)}{dy}|}_{y=1}<0$, ${\frac{d\left(F\left(y\right)\right)}{dy}|}_{y=0}>0$, it can be inferred that y=1 is the evolutionary stable point of the catering company. It shows that the caterers changed from non-intervention to intervention, and finally stabilised in the strategy of choosing intervention.

For consumers, the following conclusions can be drawn from Formula (12):

(1) When $\frac{2W-2xI_{1}-2\left(1-x\right)I_{2}}{\varepsilon f+\varepsilon i+2\mu W}<y<1$, then ${\frac{d\left(F\left(z\right)\right)}{dz}|}_{z=1}<0$, ${\frac{d\left(F\left(z\right)\right)}{dz}|}_{z=0}>0$, it can be inferred that z=1 is the evolutionary stable point of the consumer. It shows that consumers changed from waste to no waste, and finally stabilised in choosing the strategy of no waste.
(2) When $y=\frac{2W-2xI_{1}-2\left(1-x\right)I_{2}}{\varepsilon f+\varepsilon i+2\mu W}$, then F(z)≡0, It shows that consumers choose to waste and not waste the same benefits. All z are in an evolutionary stable state, and the policy choice does not change with time.
(3) When $0<y<\frac{2W-2xI_{1}-2\left(1-x\right)I_{2}}{\varepsilon f+\varepsilon i+2\mu W}$, then ${\frac{d\left(F\left(z\right)\right)}{dz}|}_{z=1}>0$, ${\frac{d\left(F\left(z\right)\right)}{dz}|}_{z=0}<0$, it can be inferred that z=0 is the evolutionary stable point of the consumer. It shows that consumers transition from no waste to waste and eventually settle on a strategy of choosing waste.

#### 3.2.2. System Stability Analysis

According to the above analysis, the eight equilibrium points of the evolutionary game system are obtained as E1(0,0,0), E2(0,0,1), E3(0,1,0), E4(1,0,0), E5(0,1,1), E6(1,0,1), E7(1,1,0) and E8(1,1,1). It is still uncertain whether the equilibrium point is asymptotically stable, and the ESS can only be achieved when both the Nash equilibrium and pure strategy Nash equilibrium are satisfied. The asymptotic stability of the equilibrium points is determined by the Lyapunov discriminant (indirect method), which first solves for the Jacobi matrix and its eigenvalues. In order to analyse the evolution and stability trend among the government, enterprises and farmers, we established the Jacobian matrix as shown in Equation (13). We obtain the eigenvalues of the Jacobi matrix by taking the first order partial derivatives of *F*(*x*), *F*(*y*) and *F*(*z*) with respect to *x*, *y* and *z*, respectively. At a certain point, if the eigenvalues of *J* are all less than 0, the equilibrium point has asymptotic stability and is an evolutionary stable point. The eigenvalues corresponding to each of the eight equilibrium points can be obtained separately by substituting them into the Jacobi matrix of Equation (13), as shown in Table 2.

(13) $$J=\left(\begin{array}{ccc} a_{11} & a_{12} & a_{13}\\ a_{21} & a_{22} & a_{23}\\ a_{31} & a_{32} & a_{33} \end{array}\right)=\left(\begin{array}{ccc} \frac{\partial F\left(x\right)}{\partial x} & \frac{\partial F\left(x\right)}{\partial y} & \frac{\partial F\left(x\right)}{\partial z}\\ \frac{\partial F\left(y\right)}{\partial x} & \frac{\partial F\left(y\right)}{\partial y} & \frac{\partial F\left(y\right)}{\partial z}\\ \frac{\partial F\left(z\right)}{\partial x} & \frac{\partial F\left(z\right)}{\partial y} & \frac{\partial F\left(z\right)}{\partial z} \end{array}\right)$$

(14) $$\left\{ \begin{array}{l} a_{11}=\frac{\partial F(x)}{\partial x}=\left(1-2x\right)[E_{g1}-E_{g2}+\beta H-y\alpha A-(1-y)\beta F]\\ a_{12}=\frac{\partial F(x)}{\partial y}=x\left(1-x\right)\left(-\alpha A+\beta F\right)\\ a_{13}=\frac{\partial F(x)}{\partial z}=0\\ a_{21}=\frac{\partial F(y)}{\partial x}=y\left(1-y\right)(\alpha A-\beta F)\\ a_{22}=\frac{\partial F(y)}{\partial y}=\left(1-2y\right)[-\mu c-ps+e+E-z\varepsilon i+(1-z)\varepsilon f+x\alpha A+(1-x)\beta F]\\ a_{23}=\frac{\partial F(y)}{\partial z}=y\left(1-y\right)(-\varepsilon f-\varepsilon i)\\ a_{31}=\frac{\partial F(z)}{\partial x}=z(1-z)(2I_{1}-2I_{2})\\ a_{32}=\frac{\partial F(z)}{\partial y}=z\left(1-z\right)\left[\varepsilon i+\varepsilon f-2(1-\mu )W+2W\right]\\ a_{33}=\frac{\partial F(z)}{\partial z}=\left(1-2z\right)[y\varepsilon i+y\varepsilon f+2xI_{1}+2(1-x)I_{2}-2y(1-\mu )W-2(1-y)W] \end{array}\right.$$

The above eight equilibrium points represent eight different situations. E1(0,0,0) indicates that under the government incentive-guided policy, all participants do not cooperate; E2(0,0,1) indicates that under the government incentive-guided policy, consumers choose to cooperate and caterers do not; E3(0,1,0) indicates that under the government incentive-guided policy, caterers choose to cooperate and consumers do not; E4(1,0,0) indicates that under the government penalty-containment policy, all participants do not cooperate; E5(0,1,1) indicates that under the government incentive-guided policy, caterers choose to intervene and consumers choose not to waste; E6(1,0,1) indicates that under the government penalty-containment policy, caterers do not cooperate but consumers choose to cooperate; E7(1,1,0) indicates that under the government penalty-containment policy, consumers do not cooperate but caterers choose to cooperate; E8(1,1,1) indicates that under the government penalty-containment policy, caterers choose to intervene and consumers choose not to waste. By comparing the actual meanings represented by the eight equilibrium points, we find that E5(0,1,1) and E8(1,1,1) represent that all participants choose to cooperate, while the other equilibrium points have not achieved cooperation, so E5 (0,1,1) and E8(1,1,1) correspond to our ideal state. According to Lyapunov’s indirect method, an equilibrium is asymptotically stable when all of its eigenvalues have negative real parts. In order to achieve these two ideal states, it is necessary to satisfy the condition that the eigenvalues of E5(0,1,1) and E8(1,1,1) are both less than 0. The specific analysis is as follows:

Case 1: (0,1,1) is the evolutionary stable point. According to Table 2, the inequality group (15) can be established. It shows that: the benefit of the government choosing punishment-inhibited policy is greater than the benefit of choosing incentive-guided policy, so the government chooses punishment-inhibited policy; the cost of non-intervention is higher than the cost of intervention, so the caterers choose to intervene; the loss of consumer waste is greater than that of non-waste losses, so consumers choose not to waste.

(15) $$\left\{ \begin{array}{l} E_{g1}+\beta H<E_{g2}+\alpha A\\ \mu c+ps+\varepsilon i<e+E+\beta F\\ 2(1-\mu )W<\varepsilon i+\varepsilon f+2I_{2} \end{array}\right.$$

Case 1 shows that under the punishment-inhibited policy, the government imposes fines on non-intervention behaviours of caterers. Moreover, caterers will not gain an environmental reputation without intervening, nor can they reduce the cost of kitchen waste disposal. Compared with the potential losses of these three parts, the cost of intervention by caterers is lower, so caterers choose to intervene. Consumers who choose not to waste under the influence of normative illusion will suffer from group exclusion, and this exclusion effect is partially offset by the correct social consumption concept established by the government’s punishment-inhibited policy. Moreover, the caterers that implemented the intervention fined the wasteful consumers and rewarded the non-wasteful consumers so that the overall cost of waste for consumers was higher than that of non-waste, so consumers choose not to waste.

Case 2: (1,1,1) is the evolutionary stable point. According to Table 2, the inequality group (16) can be established. It shows that: the benefit of the government choosing punishment-inhibited policy is lower than the benefit of choosing incentive-guided policy, so the government chooses incentive-guided policy; the cost of non-intervention is higher than the cost of intervention, so the caterers choose to intervene; the loss of consumer waste is greater than that of non-waste losses, so consumers choose not to waste. According to Table 2, the inequality group (16) can be established.

(16) $$\left\{ \begin{array}{l} E_{g2}+\alpha A<E_{g1}+\beta H\\ \mu c+ps+\varepsilon i<e+E+\alpha A\\ 2(1-\mu )W<\varepsilon i+\varepsilon f+2I_{1} \end{array}\right.$$

Case 2 shows that under the government’s incentive and guidance policy, the government rewards the intervention behaviour of caterers. In addition, caterers choose to intervene to gain an environmental reputation and reduce kitchen waste disposal costs. The benefits of intervention are greater than the costs of intervention. Therefore, caterers choose to intervene. The exclusion effect caused by normative illusion is partially offset by the correct social consumption concept established by the government’s incentive and guidance policy. Moreover, the reward and punishment measures of caterers to consumers make the overall cost of consumer waste higher than that of no waste, so consumers choose not to waste.

**Figure 1:**
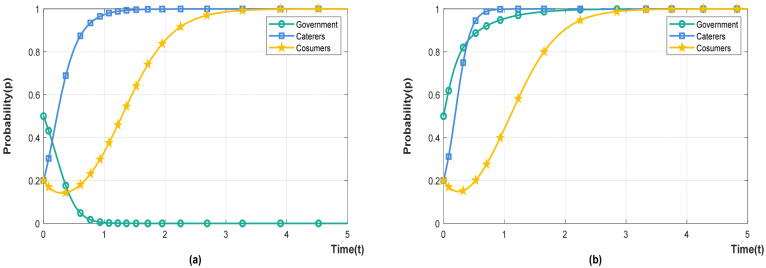
System evolution steady state. (**a**) $E_{g1}<E_{g2}$. (**b**) $E_{g1}>E_{g2}$.

A simulation of two cases is shown in Figure 1. According to a survey of caterers and consumers, there is usually no partnership between them. Therefore, it is assumed that the initial choice probability of caterers and consumers is 0.2, indicating low willingness to cooperate, and the initial probability of the government is 0.5, indicating that the initial state is not sensitive to the two policies. Keeping other conditions unchanged, the social benefits of different policies are the key factors for the government to choose corresponding policies. When $E_{g1}<E_{g2}$, the system is stable at E5(0,1,1), that is, the government chooses punishment-inhibited policy. When $E_{g1}>E_{g2}$, the system is stable at E8(1,1,1), that is, the government chooses incentive-guided policy.

## 4. Simulation Analyses of the Evolutionary Game

### 4.1. Initial Variable

This section discusses the sensitivity of key parameters in Cases 1 and 2, including the government’s incentive, punishment, caterers’ prior intervention, resultant intervention, and the probability that caterers’ intervention may lead to customer disgust. When simulating the sensitivity of a parameter, we keep other parameters unchanged. 

The initial variable of this paper was obtained through four channels. First, is the Chinese government’s policies and laws on food waste. In accordance with Article 28 of the Anti-food Waste Law of the People’s Republic of China, caterers who violate the provisions of this law and refuse to correct them shall be fined from CNY 1000 to CNY 10,000. We assume that the amount of fines imposed by the government on caterers is CNY 1000. Second, is the classic literature and China Statistical Yearbook. Under the budget, the government’s financial commitment to waste management for chefs is the same, whether it is through incentive guidance or punitive disincentives. Therefore, according to Wang, Qin [35], the government’s financial investment in managing food waste is considered to have saved the cost of environmental management of food waste because it reduces carbon emissions. Food waste per capita in the catering sector was 93 g/person/meal, and the chef waste utilisation rate represented was 11.7% [32]. Midsized restaurants have about 10 kg/day of waste. The environmental management costs per unit of carbon emissions in China can be calculated at 204.16 RMB/kg, based on official statistics from the China Statistical Yearbook, the China Environmental Statistics Yearbook and the World Bank’s total annual carbon emissions for China. Third, is research cases. According to the contact information of 10 exemplary caterers provided by the catering association of the author’s unit, the managers of these 10 caterers were interviewed in-depth. By summarising the main practices of caterers, we found that the intervention methods of caterers can be divided into three types: information prompts, rewards and punishments and food environment interventions. Among them, the cost of information prompts and rewards is estimated to be CNY 200, the penalty is CNY 100, and the cost of environmental food intervention to reduce kitchen waste in catering is estimated to be CYN 200. However, the intervention behaviour of caterers may cause dissatisfaction among consumers, causing a loss of about CYN 2000 to the caterers. Fourth, is expert opinions in the field of resources and environment. In the unequal conditions of cases 1 and 2, the initial value of interventions by satisfiers in the interests of environmental reputations, the impact on consumers of correct consumer perceptions established by governments and the parameters of consumers’ normative illusion are based on expert estimations. To ensure the scientific, accurate and effective quantification of evaluation indicators, to reduce the impact of evaluators’ subjective factors and to ensure consistency in valuation results, we recruited 12 resource and environmental experts (3 professors, 4 associate professors, 5 PhD students) and randomly assigned them to six groups. The entire valuation process consists of six steps:

1. Explaining and discussing the implications of the model structure and parameters.
2. Conducting a trial valuation and optimising the valuation criteria.
3. Conducting a pre-valuation and modifying the valuation criteria.
4. Conducting the first formal valuation.
5. Modifying the official valuation results.
6. Using the averaging method to calculate the final valuation.

The strength of government incentives and regulation and the strength of intervention by caterers are both assumed to be 0.5, indicating the government’s and caterers’ neutral attitudes towards different policies and intervention approaches in the initial state. We assume a 50% probability of consumers’ aversion to intervention by caterers. Based on information from all three sources, we simplify the data processing, as shown in Table 3.

### 4.2. Simulation of Different Policy Options

#### 4.2.1. The Influence of Government Incentives under Incentive-Guided Policy

**Figure 2:**
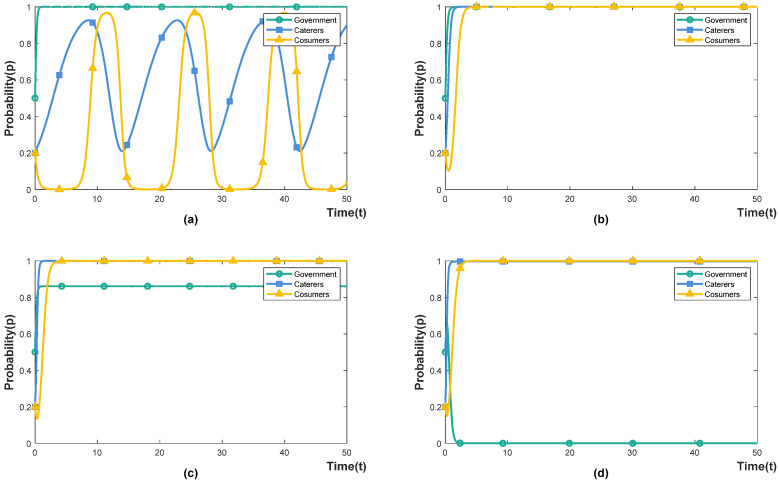
The influence of government incentives. (**a**) α=0.2. (**b**) α=0.4. (**c**) α=0.6. (**d**) α=0.8.

Under the incentive-guided policy ($E_{g1}>E_{g2}$), we set the government incentive as 0.2, 0.4, 0.6 and 0.8, indicating that government incentives account for 20%, 40%, 60% and 80% of food-waste-control fiscal expenditures, respectively. The simulation results are shown in Figure 2a–d. When the government invests 20% of fiscal expenditure, the effect of incentive-guided policies on caterers and consumers is weak. Both caterers and consumers are in a state of evolution and fluctuation, manifesting in repeated games based on their respective interests. The reason for this is that low incentives are not directly attractive to caterers, and implementing intervention may cause consumers’ disgust. For example, customers are unwilling to accept fines from caterers for wasteful behaviour. Reducing the plate size and food portion sizes can cause some customers to perceive restaurants as “unaffordable” and reduce the operating income of the caterers. Caterers can improve customer satisfaction by improving the quality of dishes or re-pricing [39]; when the government invests 40% of fiscal expenditure, caterers choose to intervene, and consumers choose not to waste. Under this incentive strength, the incentive-guided policy can effectively promote system cooperation; when the government invests 60% of fiscal expenditure, the willingness of the government to choose incentive-guided policies decreases (0.8 < *x* < 1), but caterers and consumers still choose to cooperate. The reason for this is that high incentives are more attractive to enterprises, and under the guidance of the government, the role of social norms are strengthened, reducing consumers’ normative illusion [40]; when the government invests 80% of fiscal expenditure, the government chooses to punish the containment policy, and catering companies and consumers still choose to cooperate. It shows that high incentives have caused a heavy financial burden, and the government chooses to implement a punishment-inhibited policy to reduce fiscal expenditures.

#### 4.2.2. The Influence of Government Punishment under Punishment-Inhibited Policy 

**Figure 3:**
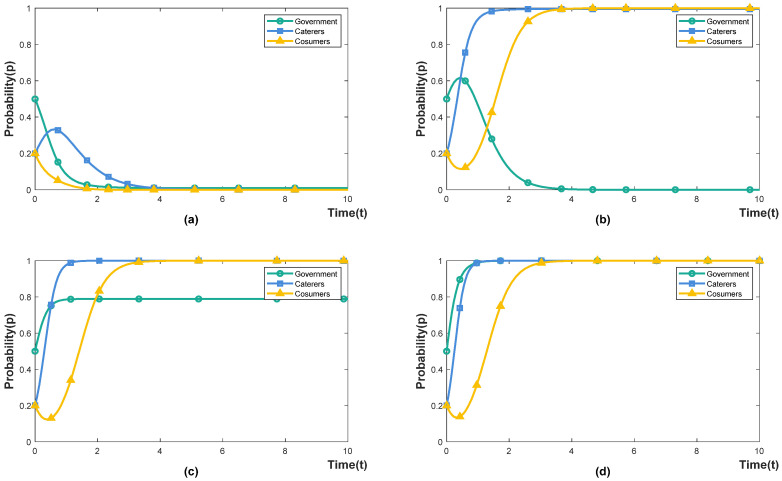
The influence of government punishment. (**a**) β=0.2. (**b**) β=0.5. (**c**) β=0.6. (**d**) β=0.8.

Under the punishment-inhibited policy ($E_{g1}>E_{g2}$), we set the government punishment (or supervision) as 0.2, 0.5, 0.6 and 0.8, which means that the cost of government supervision accounts for 20%, 50%, 60% and 80% of the food-waste-control fiscal expenditure, respectively. The simulation results are shown in Figure 3a–d. When the government chooses a punishment-inhibited policy, it must pay the supervision cost, and the supervision intensity is positively correlated with the punishment intensity. When the government invests 20% of fiscal expenditure, the government imposes fewer fines on caterers. In order to avoid the possibility of consumer disgust caused by the intervention, caterers choose not to intervene. China’s long-term “wine table and banquet culture” has given birth to the wrong concept of consumption that is proud of waste and ashamed of thrift. Under the influence of wrong food consumption concepts, consumers believe extravagance and waste are “face-saving” behaviours. If caterers do not intervene, consumers’ wasteful behaviour cannot be corrected in time [27]; when the government invests 50% of fiscal expenditure, both caterers and consumers choose to cooperate. The reason for this is that increasing the punishment increases the certainty cost of the non-intervention behaviour of caterers, which makes the attitude of caterers change from non-intervention to intervention. The intervention behaviour of caterers reduces consumers’ normative illusion and increases the cost of waste for consumers so that consumers choose not to waste [41]; when the fiscal expenditure invested by the government is 60% or greater, both caterers and consumers choose to cooperate. However, at this time, the government’s willingness to choose a punishment-inhibited policy is declined. The reason for this is that the high supervision cost has increased the government’s financial burden, and the government has chosen a lower investment incentive-guided policy.

According to the above analysis, both incentive-guided policy and punishment-inhibited policy can enable caterers and consumers to participate in reducing food waste. However, the implementation of a punishment-inhibited policy requires more financial investment. To achieve this ideal effect, the condition set in the game model must be satisfied. The government’s implementation of incentive-guided policies can effectively establish social conservation awareness and reduce consumers’ normative illusion. In reality, the amount of incentive-guided policies is not attractive to caterers. For example, the incentive policies in the United States to guide social subjects to donate food to non-profit organisations have little effect [42]. On the contrary, under the punishment-inhibited policy, caterers that do not intervene will be punished with administrative punishments, such as circular criticism, and ordering rectification will bring greater tangible losses to the reputation of caterers. Therefore, the willingness of enterprises to intervene is stronger under the punishment-inhibited policy [43]. 

### 4.3. Improvements to Penalty Containment Policy

According to the policy simulation results in Figure 2 and Figure 3, the implementation effect of the punishment-inhibited policy is not ideal. This section explores how the government’s punishment-inhibited policy can be improved by other means.

#### 4.3.1. The Effect of Prior Intervention *μ* on the Punishment-Inhibited Policy 

**Figure 4:**
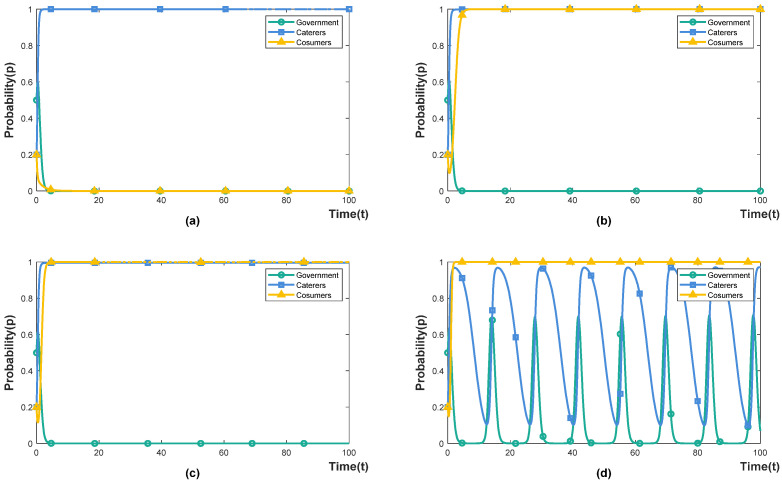
The effect of μ on the evolution trajectory. (**a**) μ=0.2. (**b**) μ=0.4. (**c**) μ=0.6. (**d**) μ=0.8.

Under the government’s punishment-inhibited policy ($E_{g1}<E_{g2}$), we set the strength of the prior intervention for caterers to be 0.2, 0.4, 0.6 and 0.8, respectively, indicating that the actual expenditure of the prior intervention accounts for 20%, 40%, 60% and 80% of the cost of the caterers’ prior intervention. The simulation results are shown in Figure 4. When caterers invest 80% of the cost of prior intervention, both the caterers and the government are in a state of evolution and fluctuation, and the punishment-inhibited policy fails; when caterers invest 40–60% of the cost of prior intervention, the government’s implementation of punishment-inhibited policy can make the system an ideal state (0,1,1). When the cost of prior intervention invested by caterers is less than 20%, consumers will choose to waste even if caterers participate in cooperation. The reason for this is that establishing good social consumption habits requires a high degree of persistence to instill the concept of rational consumption and food saving in public, and cannot rely solely on short-term means such as propaganda boards or prompts [44]. However, high intervention intensity increases the burden on caterers and reduces the willingness to intervene.

#### 4.3.2. The Effect of the Probability *p* of Consumer Dissatisfaction on the Punishment-Inhibited Policy 

**Figure 5:**
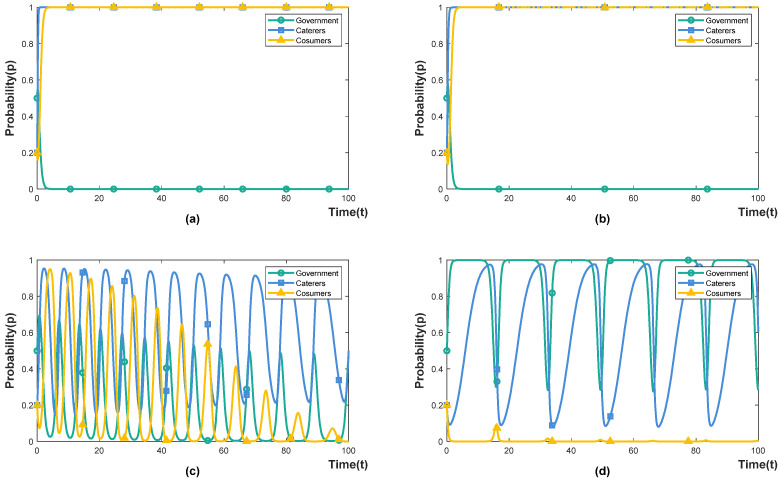
The effect of p on the evolution trajectory. (**a**) p=0.2. (**b**) p=0.4. (**c**) p=0.6. (**d**) p=0.8.

Under the government’s punishment-inhibited policy ($E_{g1}<E_{g2}$), we set the probability that consumers are dissatisfied with the caterers’ intervention as 0.2, 0.4, 0.6 and 0.8, respectively, representing the proportion of consumers who are dissatisfied with the caterers’ intervention. The simulation results are shown in Figure 5. When the probability of consumers being dissatisfied with the intervention of caterers is less than 40%, the government’s punishment-inhibited policy can stabilise the system in an ideal state (0,1,1); When the probability of consumer dissatisfaction is equal to or greater than 60%, the entire system is in a state of evolutionary fluctuation. With the increase in evolution time, the probability of consumers choosing not to waste is decreasing, and the government’s penalty containment policy fails. It shows that when the probability of consumer dissatisfaction is lower than 40%, the intervention behaviour of caterers and the non-waste behaviour of consumers can achieve a virtuous circle. On the one hand, the intervention behaviour of caterers has improved the public’s awareness of saving and reduced consumers’ normative illusion of being “proud of extravagance and waste” [19]. On the other hand, consumers with a sense of saving are less likely to be disgusted by the intervention of caterers, increasing their confidence in their intervention [45]. The probability of consumers’ dissatisfaction with caterers is affected by their intervention methods. Compared with prior intervention, implementing resultant interventions and food environment interventions by caterers is more likely to reduce consumer satisfaction [46]. 

#### 4.3.3. The Effect of Resultant Interventions ε on the Punishment-Inhibited Policy 

**Figure 6:**
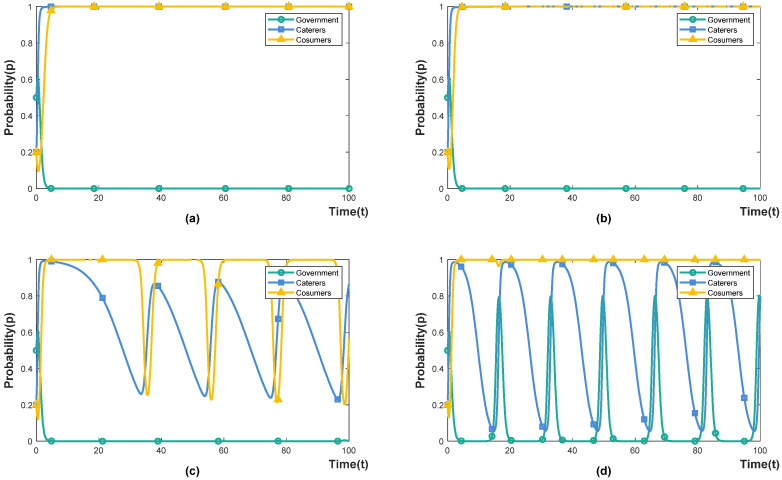
The effect of ε on the evolution trajectory. (**a**) ε=0.2. (**b**) ε=0.4. (**c**) ε=0.6. (**d**) ε=0.8.

Under the government’s punishment-inhibited policy ($E_{g1}<E_{g2}$), we set the intensity of the caterers’ implementation of resultant intervention as 0.2, 0.4, 0.6 and 0.8, indicating that the actual expenditure of the resultant intervention accounts for 20%, 40%, 60% and 80% of the cost of the caterers’ resultant intervention, respectively. The simulation results are shown in Figure 6. The resultant interventions include rewards and punishments for consumer behaviour. When consumers choose to waste, caterers impose fines on them, and when consumers choose not to waste, caterers reward them. Under the government’s punishment-inhibited policy, the system reaches an ideal stable state (0,1,1) when caterers invest 40% of the cost of resultant intervention. When caterers invest 60% of the cost of resultant intervention, the whole system is in a state of evolution and fluctuation, and the government’s punishment-inhibited policy fails. It shows that caterers only need to implement low incentives (ε≤0.4) to make consumers participate in cooperation. High penalties (ε=0.6) will arouse consumers’ disgust and increase the uncertain cost of caterers, while high penalties and high rewards lead to consumers not cooperating. Therefore, under the punishment-inhibited policy, caterers can encourage consumers to participate in cooperation by formulating a reasonable reward and punishment system, or by substituting rewards for punishment.

## 5. Discussion

According to statistics, 1.3 billion tons of food are wasted globally annually, causing direct economic losses of more than USD 940 billion [47]. Food waste at the consumer end has become a global problem. The US has enacted the Bill Emerson Good Samaritan Food Donation Act, Internal Revenue Code and the U.S. Federal Food Donation Act to encourage organisations and individuals to donate food to non-profits. The European Commission issued the Waste Framework Directive in 2008 and established the Waste Prevention Programmers (WPMs). In 2016, the French government enacted the Anti-Food Waste Act, and in 2018, amendments were made to the Agriculture and Food Act. In order to reduce food losses, Japan passed legislation and launched a nationwide campaign called No-Food Loss Project. Despite the ‘CD-ROM campaign’ being strongly promoted in China as early as 2013, the Anti-Food Waste Law of the People’s Republic of China did not become law until 2021 [48]. In some studies, government involvement is shown to help reduce food waste [20,49,50]. Relevant policies are mainly divided into incentive-guided and punishment-inhibited types. Among them, the main function of the incentive-guided policy is to reduce the uncertainty cost of enterprises intervening in consumers’ wasteful behaviour through transfer payment. Incentive-guided policies are implemented in the United States, Japan and Italy. The main function of the punishment-inhibited policy is to increase the deterministic cost of enterprises’ non-intervention of consumers’ wasteful behaviour. France and China are implementing punishment-inhibited policies. The objective of both policies is to engage caterers in food waste management through incentives and compulsion, creating a demonstration effect and shaping better social consumption norms. Anti-food waste policies need to be tailored to the national context. In China, food waste at the table is influenced by a ‘shame culture’, so reducing consumer illusions and encouraging caterers to participate in governance is key to policymaking. Compared to punishment-inhibited policies, incentive-guided policies have a better implementation effect. When the current regulation effect is not ideal, improving the punishment-inhibited policy can also achieve an ideal state for caterers and consumers. Policy simulations are virtual imitations of the effects of implementing policy instruments in the real world. Since they are future-oriented, their results cannot be verified by traditional out-of-sample fittings [51]. Nevertheless, research on government policies has been found to support the claims in this paper [43,52,53,54]. First, the government can utilise publicity and incentives to encourage compliance with social norms, whereas punitive measures may be counterproductive. Second, in reality, incentive policies often work very slowly, and punitive policies often serve as a quick warning, up to a point.

Consumer behaviour is a key element of food waste management, as it is affected by many factors, such as dietary habits, cultural customs, policies, and regulations. Social norms derived from these factors are crucial to restraining consumer waste behaviour [41]. In Chinese society, a sense of collectivism makes them good communicators, and waste has a strong cultural background at the dinner table [27]. Until 2021, the Chinese government had not issued legally binding documents on food waste but encouraged people to save food through advocacy and publicity. Despite this, as people’s living standards have increased, food waste has not been solved. China’s anti-food waste law was officially implemented in April 2021, which enabled the country to establish good food consumption concepts through learning, demonstrations and warnings. However, consumers’ food waste behaviour may also result from the difference between their perceived consumption norms and the group norm. The caterers’ interventions, which are directly linked to consumers eating out, can help reduce consumers’ food waste behaviour. Studies highlighted the need for more interventions to stop and change consumers’ food waste behaviour [55], and that the appropriate interventions can reduce food waste in the catering sector by 30–50% [56]. Caterers can increase consumers’ motivation to reduce waste with promotional tips, coupons, and discounts. Still, Chinese caterers mostly resort to fines for leftovers after meals due to business costs, resulting in ineffective interventions by caterers. Furthermore, Chinese caterers rarely list the number of dishes, which results in an information asymmetry between consumers and the caterers. Customers order meals based on experience rather than appetite, resulting in food waste.

## 6. Conclusions and Policy Implications

### 6.1. Conclusions

Based on evolutionary game theory, this paper constructs a tripartite evolutionary game model of the government, caterers and consumers. We simulate the different government policies and the intervention methods of caterers, revealing the impact of government policies and caterers’ interventions on the consumer who wastes with normative illusion. The main conclusions are as follows:

First, when μc+ps+εi<e+E+βF and $2\left(1-\mu \right)W<\varepsilon i+\varepsilon f+I_{2}$ or μc+ps+εi<e+E+αA and $2\left(1-\mu \right)W<\varepsilon i+\varepsilon f+I_{1}$ are satisfied, food waste can be reduced by incentive-guided and punishment-inhibited policies. Additionally, incentive-guided policies can reduce government expenditures more than punishment-inhibited ones. Second, implementing the prior intervention method, the resultant intervention method and reducing the probability of consumers’ aversion to the intervention behaviour of caterers can optimise the government’s punishment-inhibited policy. Third, under the punishment-inhibited policy, caterers can bear 60% of the prior intervention costs for food waste management. When caterers invest 40–60% of the prior intervention costs, both caterers and consumers can achieve the ideal state of cooperation; caterers can accept 40% of the resultant intervention cost for food waste management, and when the resultant intervention cost is less than 40%, consumers choose not to waste. When the resultant intervention cost is higher than 40%, it will increase the cost burden of caterers and cause consumers to be disgusted with the excessive intervention of caterers; both caterers and consumers are involved in reducing food waste when the probability of consumer dissatisfaction with a caterer’s intervention is reduced to less than 40%.

### 6.2. Policy Implications

Based on the above analysis conclusions, we propose the following three policy recommendations.

First, is the strengthening of publicity and education, creating a new trend of diligence and thrift. The government should: take more steps to educate the public and promote the consumption concept of “civilised, healthy, rational, and green”; incorporate food nutrition education into the national education system, and infiltrate the concept of diligence and thrift into the basic education of young people; enhance normalised publicity of thrift and avoidance of waste, making diligence and thrift a common social behaviour, and reducing consumers’ cognitive illusion of norms. Secondly, catering innovation should be encouraged to guide the implementation of healthy and civilised consumption; encourage caterers to enrich the variety of dishes and improve the quality; under the premise of meeting the individual needs of consumers, appropriately adjust the menu capacity, provide consumers with “half dishes” and “small dishes” in the form of dishes, and standardise and indicate the number of dishes; caterers should provide mandatory training to service staff. When consumers order food, the service staff should remind them to order according to the number of diners and dishes. After consumers dine, service staff should take the initiative to provide environmentally friendly lunch boxes to help them pack to cultivate consumers’ habit of packing leftovers. Finally, improve the system construction and formulate detailed rules for implementing laws and policies. Currently, most of China’s anti-food waste policy is only a moral regulation, and how the appropriate incentives, penalties and tax policies are formulated and implemented has not yet been clarified. The government should strengthen food waste legislation, supervision and inspection, scientific research and platform support in food consumption and waste, and formulate detailed rules for implementing laws and policies.

### 6.3. Limitations

We recognise and acknowledge the existence of some important limitations due to basic model assumptions, which may provide avenues for future research. First, the focus on food and beverage waste in this paper limits the scope of the findings, and the relationship between food waste behaviour occurring within the household context and consumer normative illusions still requires further analysis. Second, this paper is only based on the government, caterers and consumers and does not consider the influence of other stakeholders. Future research on food waste could further expand the scope of the study to consider the impact of different stakeholders on reducing food waste. Third, the construction of the model ignores the heterogeneity of individual behaviour and many psychological factors. Subsequent research could focus on individual differences, such as consumers’ education levels, and irrational factors, such as loss aversion and risk aversion of restaurant companies to implement interventions to construct a more realistic model of food waste evolution.

## Figures and Tables

**Table 1:** Players’ payoff matrix.

		Consumers	Government	
			Incentive-Guided (x)	Punishment-Inhibited (1−x)
Caterers	Intervene (y)	Not waste (z)	$-\alpha A+E_{g1},$ −μc−εi−ps+e+E+α, $\varepsilon i-\left(1-\mu \right)W+I_{1}$	$-\beta H+E_{g2},$ −μc−εi−ps+e+E, $\varepsilon i-\left(1-\mu \right)W+I_{2}$
		Waste(1−z)	$-\alpha A+E_{g1}-G,$ −μc+εf+e+E−ps+αA, $-\varepsilon f+\left(1-\mu \right)W-I_{1}$	$-\beta H+E_{g2}-G,$ −μc+εf+e+E−ps $,-\varepsilon f+\left(1-\mu \right)W-I_{2}$
	Not intervene (1−y)	Not waste (z)	$E_{g1},$ 0, $-W+I_{1}$	$-\beta H+E_{g2}+\beta F,$ −βF, $-W+I_{2}$
		Waste (1−z)	$-G+E_{g1},$ 0, $W-I_{1}$	$-\beta H+\beta F+E_{g2}-G,$ −βF, $W-I_{2}$

**Table 2:** Stability of equilibrium points.

Equilibrium	Jacobian Eigenvalues
	$\lambda_{1}$ $,\;\;\lambda_{2}$ $,\;\;\lambda_{3}$
E1(0,0,0)	$E_{g1}-E_{g2}+\beta H-\beta F$ ,−μc−ps+e+E+εf+βF $,2I_{2}-2W$
E2(0,0,1)	$E_{g1}-E_{g2}+\beta H-\beta F$ ,−μc−ps+e+E−εi+βF $,2W-2I_{2}$
E3(0,1,0)	$E_{g1}-E_{g2}+\beta H-\alpha A$ ,μc+ps−e−E−εf−βF $,\varepsilon i+\varepsilon f-2\left(1-\mu \right)W+2I_{2}$
E4(1,0,0)	$-E_{g1}+E_{g2}-\beta H+\beta F$ ,−μc−ps+e+E+εf+αA $,2I_{1}-2W$
E5(0,1,1)	$E_{g1}-E_{g2}+\beta H-\alpha A$ ,μc+ps−e−E+εi−βF $,-\varepsilon i-\varepsilon f+2\left(1-\mu \right)W-2I_{2}$
E6(1,0,1)	$-E_{g1}+E_{g2}-\beta H+\beta F$ ,−μc−ps+e+E−εi+αA $,2W-2I_{1}$
E7(1,1,0)	$-E_{g1}+E_{g2}-\beta H+\alpha A$ ,μc+ps−e−E−εf−αA $,\varepsilon i+\varepsilon f-2\left(1-\mu \right)W+2I_{1}$
E8(1,1,1)	$-E_{g1}+E_{g2}-\beta H+\alpha A$ ,μc+ps−e−E+εi−αA $,-\varepsilon i-\varepsilon f+2\left(1-\mu \right)W-2I_{1}$

**Table 3:** The initial values of all variables (unit: CNY 10^2^).

Participants	Parameters	Variables	Value
Government	Government oversight costs	H [35,36,37]	20
	Rewards to caterers for implementing the intervention	A [35,36,37]	20
	Failure to intervene leads to fines for caterers	F [38]	10
Caterers	Caterers’ losses due to customer dissatisfaction	s (Interview)	20
	The cost of prior intervention for caterers	c (Interview)	2
	Rewards from caterers to consumers	i (Interview)	2
	Fines from caterers to consumers	f (Interview)	1
	Intervene in the food environment to save the cost of kitchen waste disposal in catering	e (Interview)	2
	Environmental reputation benefits	E (Expert valuation)	5
Consumers	Under the government’s incentive and guidance policy, the correct consumption concept brings benefits for consumers not to waste	$I_{1}$ (Expert valuation)	3
	Under the government’s punishment and containment policy, the correct consumption concept brings benefits for consumers not to waste	$I_{2}$ (Expert valuation)	3
	Normative illusion benefits	W (Expert valuation)	5

## Data Availability

The specific data of the survey samples cannot be made public.

## Nomenclature

α	Government’s incentives intensity
β	Government’s oversight intensity
μ	The intensity to implement prior interventions
ε	The intensity to implement resultant interventions
p	The probability of customer disgust caused by the intervention of caterers
H	Government oversight costs
G	Government losses due to wasting food
A	Rewards to caterers for implementing the intervention
F	Failure to intervene leads to fines for caterers
$E_{g1}$	The social benefits obtained by the government’s implementation of incentive-guided policy
$E_{g2}$	The social benefits obtained by the government’s implementation of punishment-inhibited policy
c	The cost of prior intervention for caterers
i	Rewards from caterers to consumers
f	Fines from caterers to consumers
e	Intervene in the food environment to save the cost of kitchen waste disposal in cates
s	Caterers’ losses due to customer dissatisfaction
E	Environmental reputation benefits
W	Normative illusion benefits
$I_{1}$	Under the government’s incentive-guided policy, the correct consumption concept brings benefits for consumers not to waste
$I_{2}$	Under the government’s punishment-inhibited policy, the correct consumption concept brings benefits for consumers not to waste
